# Aminoglucose-functionalized, redox-responsive polymer nanomicelles for overcoming chemoresistance in lung cancer cells

**DOI:** 10.1186/s12951-017-0316-z

**Published:** 2017-11-28

**Authors:** Yi Zhou, Huaying Wen, Liang Gu, Jijun Fu, Jiayi Guo, Lingran Du, Xiaoqin Zhou, Xiyong Yu, Yugang Huang, He Wang

**Affiliations:** 1 Key Laboratory of Molecular Clinical Pharmacology & Fifth Affiliated Hospital, Guangzhou Medical University, Guangzhou, 511436 Guangdong China; 2 Center of Cancer Research, the Second Affiliated Hospital, Guangzhou Medical University, Guangzhou, 510260 Guangdong China

**Keywords:** Gult-1, Redox-responsive polymer, Nanomicelles, Multidrug resistance, Cancer therapy

## Abstract

**Background:**

Chemotherapeutic drugs used for cancer therapy frequently encounter multiple-drug resistance (MDR). Nanoscale carriers that can target tumors to accumulate and release drugs intracellularly have the greatest potential for overcoming MDR. Glucose transporter-1 (GLUT-1) and glutathione (GSH) overexpression in cancer cells was exploited to assemble aminoglucose (AG)-conjugated, redox-responsive nanomicelles from a single disulfide bond-bridged block polymer of polyethylene glycol and polylactic acid (AG-PEG-SS-PLA). However, whether this dual functional vector can overcome MDR in lung cancer is unknown.

**Results:**

In this experiment, AG-PEG-SS-PLA was synthetized successfully, and paclitaxel (PTX)-loaded AG-PEG-SS-PLA (AG-PEG-SS-PLA/PTX) nanomicelles exhibited excellent physical properties. These nanomicelles show enhanced tumor targeting as well as drug accumulation and retention in MDR cancer cells. Caveolin-dependent endocytosis is mainly responsible for nanomicelle internalization. After internalization, the disulfide bond of AG-PEG-SS-PLA is cleaved in the presence of high intracellular glutathione levels, causing the hydrophobic core to become a polar aqueous solution, which subsequently results in nanomicelle disassembly and the rapid release of encapsulated PTX. Reduced drug resistance was observed in cancer cells in vitro. The caspase-9 and caspase-3 cascade was activated by the AG-PEG-SS-PLA/PTX nanomicelles through upregulation of the pro-apoptotic proteins Bax and Bid and suppression of the anti-apoptotic protein Bcl-2, thereby increasing apoptosis. Furthermore, significantly enhanced tumor growth inhibition was observed in nude mice bearing A549/ADR xenograft tumors after the administration of AG-PEG-SS-PLA/PTX nanomicelles via tail injection.

**Conclusions:**

These promising results indicate that AG-PEG-SS-PLA/PTX nanomicelles could provide the foundation for a paradigm shift in MDR cancer therapy.

**Electronic supplementary material:**

The online version of this article (10.1186/s12951-017-0316-z) contains supplementary material, which is available to authorized users.

## Background

Multidrug resistance (MDR) is a major impediment to the clinical success of chemotherapy and leads to low clinical anticancer efficacy [1, 2]. P-glycoprotein (P-gp) is often overexpressed in the plasma membrane of many MDR cells, and it mediates drug efflux, which might represent an important mechanism of the resistance of these cells to various anticancer drugs [3]. Many nanoscale drug delivery systems have been used for the inhibition of drug efflux mediated by P-gp [4]. For example, Feng and Mei reported that drug-loaded nanocarriers based on d-α-tocopheryl polyethylene glycol 1000 succinate (TPGS) could improve cancer chemotherapy against MDR [5, 6]. In addition, copolymer nanomicelles whose surfaces are functionalized with tumor-specific targeting moieties (e.g., receptor-binding ligands or antibodies) can bypass P-gp to kill MDR tumor cells via increased drug concentrations in tumors; thus, they are considered to be active targeting drug delivery systems for tumor treatment [7].

Glucose uptake in tumors is substantially higher than that in adjacent normal tissues [8, 9]. However, glucose requires specific transport proteins to enter the cytosol [10], and the transportation of glucose into cancer cells is mainly mediated by facilitative glucose transporters (GLUT) [11, 12]. GLUT-1 is the most studied glucose transporter and likely represents a driver of glucose uptake in cancer cells. Although GLUT-1 is expressed in normal tissues, particularly erythrocytes, binding assays of glucose-functional nanomaterials have not revealed significant binding between GLUT-1 and ovine red blood cells or hemolysis [13], which indicates that targeting tumors using a GLUT-1 media preparation is less toxic than traditional therapies. Moreover, the overexpression of GLUT-1, which has been verified in a variety of cancer cells, including lung cancer cells [8], has been a target of drug treatment.

The intracellular accumulation of encapsulated drugs is a characteristic of nanocarriers that can be used to overcome MDR via the endocytosis pathway [14]. However, insufficient drug release in tumor cells could inhibit drug accumulation and lead to concentrations below the therapeutic threshold [15]. Particular attention has been focused on stimulus–sensitive drug delivery systems that can release encapsulated drugs via an intracellular stimulus trigger, such as pH, redox, or specific enzymes [16, 17]. Specifically, drug delivery systems containing disulfide linkages, which can be reduced to thiol groups by glutathione (GSH), are potential platforms for overcoming drug resistance through their rapid intracellular drug delivery due to the large difference between the GSH concentrations in the cell exterior (∼ 2 μM) and interior (∼ 10 mM) [18].

**Scheme 1:**
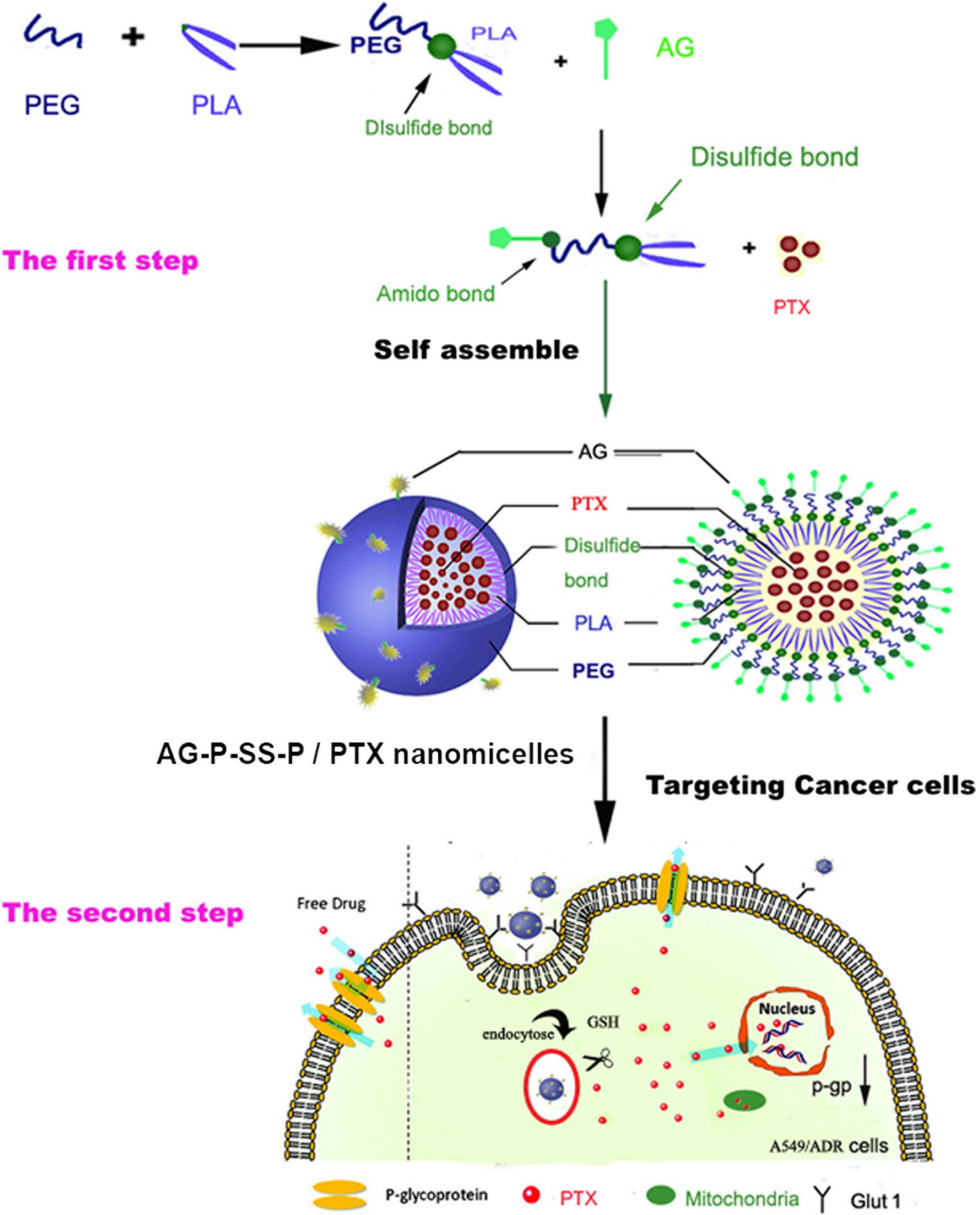
Schematic representation of the assembly, tumor targeting, and cellular internalization of AG-PEG-SS-PLA nanomicelles leading to reversal of MDR in lung cancer therapy

Paclitaxel is a natural plant product extracted from the bark of western *Taxus brevifolia*, and it is active against a broad range of cancers mainly by acting on the microtubules of cancer cells [19]. Paclitaxel can also act directly on isolated mitochondria from cancer cells as well as on mitochondria in intact cells, resulting in the apoptosis of cancer cells [20–22]. However, the poor aqueous solubility of PTX significantly limits its anticancer activity [23]. Here, we introduce a dual-function nano drug delivery system consisting of redox-responsive nanomicelles based on a disulfide-bond-linked block polymer of polyethylene glycol (PEG) and polylactic acid (PEG-SS-PLA) that can perform targeted delivery of PEG-SS-PLA drug-loaded vehicles to target cells and tissues via glucosamine (AG) modified to PEG-s-s-PLA (AG-PEG-SS-PLA, AG-P-SS-P). These vesicles were engineered for specific tumor cells with a redox-responsive property that allows the targeting of tumor cells via GLUT-1 and the regulation of drug release via glutathione. This dual-function behavior was then harnessed to overcome the drug resistance of cancer cells using paclitaxel (PTX)-resistant A549 lung cells as a model (Scheme 1).

## Methods

### Materials and general characterization

HO-PLA-COOH (*Mw* = 13,500) was purchased from the Shandong Institute of Medical Instruments (Jinan City, Shandong Province, China), and allyloxy polyethylene glycol (APEG, *Mw* = 2400) was purchased from Jiaxing Bomei Biotech Co., Ltd. (Jinan City, Shandong Province, China). Cystamine dihydrochloride (cystamine·2HCl), *p*-nitrophenyl chloroformate (p-NPC), succinic anhydride (99%), and thioglycolic acid were purchased from Alfa Aesar (Shanghai City, China). Pyrene and reduced GSH (purity = 98%) were purchased from Aladdin Chemical Company, Ltd. (Shanghai, China). Sodium cholate, filipin, amiloride, thioglycolic acid, rhodamine 123 (Rh123), anhydrous tetrahydrofuran (THF), and AG were purchased from Sigma-Aldrich (St. Louis, MO, USA). Fetal bovine serum (FBS) and RPMI 1640 medium were purchased from HyClone and Gibco, respectively (Thermo Fisher Scientific Co., Ltd, Shanghai City, China). The size and size distribution of the nanomicelles were analyzed using a Malvern Zetasizer Nano ZS90 instrument (Malvern Instruments, Worcestershire, UK). The concentration of PTX was determined by high-performance liquid chromatography (Waters Corp., Waltham, MA, USA). The morphology of the nanomicelles was analyzed with a JEOL JEM2010 (JEOL, Tokyo, Japan) transmission electron microscope (TEM).

### Synthesis of AG-P-SS-P

#### Synthesis of the COOH-P-SS-P copolymer

APEG-SS-NH_2_ was synthesized according to the procedure reported by Zhang et al. [24]. APEG was activated with p-NPC and reacted with cystamine dihydrochloride in the presence of Et_3_N. First, a solution of p-NPC (3.21 g, 16 mmol) in 20 mL of dichloromethane (DCM) was added dropwise to a DCM solution (50 mL) of APEG (9.6 g, 4 mmol) and pyridine (0.81 mL, 10 mmol) at 0 °C for 24 h. This solution was subsequently added dropwise to a DCM solution (50 mL) of cystamine dihydrochloride (2.89 g, 12.88 mmol) and Et_3_N (4.21 mL, 30 mmol) at room temperature (RT). The entire reaction process was performed under a nitrogen atmosphere. The reaction was allowed to continue for 24 h. The resulting APEG-SS-NH_2_ was isolated by two precipitation processes in ether and then dialyzed [molecular weight cut-off (MWCO) = 3500 Da] against deionized water for 3 days at room temperature to remove the unreacted cystamine dihydrochloride.

APEG-SS-PLA was synthesized according to the procedure reported by Sun et al. [25]. Briefly, 7.14 g COOH-PLA-OH (0.50 mmol) was dissolved in 20 mL of DCM in a flask, and 140 mg of ethylene dichloride (EDC) was added under stirring at RT. After 0.5 h, 1 g of APEG-SS-NH_2_ (0.5 mmol), which had been dissolved in 5 mL of DCM, was added under stirring. The mixture was then incubated with stirring at RT for 24 h. The product was collected by dropping the reacting complexes into excess anhydrous diethyl ether to remove the unreacted APEG-SS-NH_2_. APEG-SS-PLA was collected by centrifugation and dried under vacuum for 72 h.

To synthesize COOH-PEG-SS-PLA via the thiol-ene reaction, 3.20 g of APEG-SS-PLA (0.20 mmol), 0.20 g of thioglycolic acid (0.24 mmol), and 23 mg of DMPA (0.09 mmol) were dissolved in 5 mL of THF. The product was precipitated in diethyl ether to yield a white powder.

#### Synthesis of the AG-P-SS-P copolymer

**Scheme 2:**
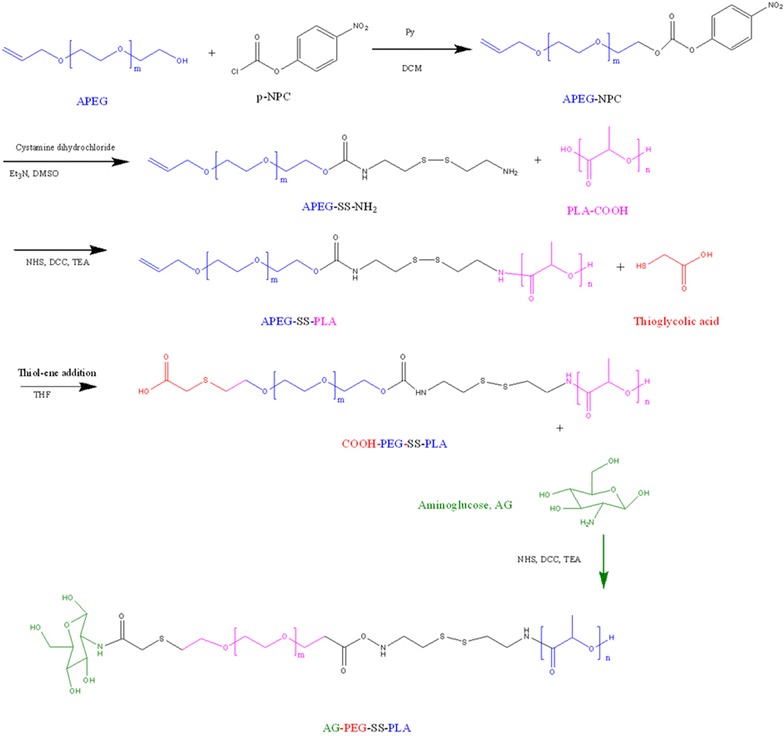
Synthetic scheme showing the various steps required to prepare AG-PEG-SS-PLA

The obtained product, COOH-PEG-SS-PLA (1.6 g, 0.1 mM), was dissolved in a solution of AG (50 mg, 0.27 mM), DCC (40 mg), NHS (20 mg), and triethylamine (0.2 mL) in DCM (10 mL) and stirred for 24 h at room temperature. The mixture was then dialyzed (MWCO = 3500 Da) against deionized water for 48 h. The final solution was lyophilized and stored at – 20 °C until use (Scheme 2).

To demonstrate disassembly of the AG-P-SS-P nanomicelles in response to GSH, the fluorescent molecule pyrene was used as a probe. The concentration of pyrene in acetone in the ampules was 10.0 μL (0.06 mM). After the acetone was volatilized, solutions of AG-P-SS-P nanomicelles (1.0 mL, 1.0 mg/mL) were added to the ampules. The final concentration of pyrene in each flask was 6.0 × 10^−7^ mol/L, and GSH was then added to the micelle solution at a final concentration of 10 mM. At different time points, the excitation spectra of pyrene were recorded using a spectrofluorophotometer (Shimadzu RF-5301PC, Kyoto, Japan).

### Preparation of PTX-loaded AG-P-SS-P (AG-P-SS-P/PTX) nanomicelles

PTX-loaded nanomicelles were prepared using a previously reported method [26]. Briefly, 10 mg of PTX (Nanjing Tianzun Zezhong Chemicals, Co., Ltd., Nanjing, China) were dissolved in anhydrous dimethyl sulfoxide (DMSO; 10 mL). After adding AG-P-SS-P (100 mg) to the solution, the mixture was stirred overnight, transferred to a wet dialysis tube (MWCO = 1 kDa) and maintained at room temperature for 24 h. The final product was filtered with a syringe filter (0.45 mm, Millipore) and then used for in vitro and in vivo studies. P-P/PTX and P-SS-P/PTX were prepared using the same procedure. The particle size and zeta potential of the nanoparticles were characterized using a Malvern Zetasizer Nano ZS90.

### In vitro AG-P-SS-P/PTX drug release measured by HPLC

The prepared AG-P-SS-P/PTX nanomicelles were suspended at a PTX concentration of 50 μg/mL in phosphate buffer (PB, 0.02 M, pH 7.4). After the solution (1 mL) was transferred to dialysis tubing, PB (10 mL) or PB with GSH (10 mM) was added to immerse the tubing. At predetermined intervals, the external buffer was collected, and an equivalent volume of fresh buffer was added. The concentration of PTX in the collected solution was determined by HPLC analysis [27]. The drug-loading capacity (DLC) and drug-loading efficiency (DLE) were calculated according to the following formulas:

$$ {\text{DLC}}\;\left( \% \right)  = \left[ {{\text{weight}}\;{\text{of}}\;{\text{drug}}\;{\text{used}}/\left( {{\text{weight}}\;{\text{of}}\;{\text{polymer }} + {\text{ drug}}\;{\text{used}}} \right)} \right] \times  100\% $$

$$ {\text{DLE}}\;\left( \% \right)  = \left( {{\text{weight of loaded drug}}/{\text{weight of input drug}}} \right)  \times 100\%$$

### Cell culture

Human lung adenocarcinoma A549 cells (College of Pharmaceutical Science, Guangzhou Medical University, Guangzhou, China) were grown in RPMI-1640 (Macgene Biotech Co., Ltd., Beijing, China) supplemented with 10% fetal bovine serum and 1% antibiotics (100 U/mL penicillin and 100 mg/mL streptomycin). The drug-resident human lung cancer cell line A549 (A549/ADR; College of Pharmaceutical Science, Guangzhou Medical University, Guangzhou, China) with P-gp overexpression was cultured in RPMI 1640 with 10% FBS and 1% antibiotics (100 U/mL penicillin and 100 mg/mL streptomycin) (Additional file 1: Figure S1). For the maintenance of drug resistance, A549/ADR cells were cultured in the presence of 4 µM CDDP, and CDDP-free medium was used for 1 week prior to the initiation of the experiments [20]. Cell cultures were performed in a humidified incubator maintained at 37 °C containing 5% CO_2_. All of the cell studies were approved by the Institutional Animal Care Committee and the Local Veterinary Office and Ethics Committee at Guangzhou Medical University (GZMUC 10-05010).

### Cellular uptake and cellular uptake mechanism

After the A549/ADR cells were cultured in 24-well plates (1 × 10^5^ cells per well) for 24 h, the medium was replaced with or without AG-P-SS-P/Rh123 nanomicelles with GSH (10 mM) for 8 h. To investigate cellular uptake and its mechanism, free Rh123, P-P/Rh123, P-SS-P/Rh123, or AG-P-SS-P/Rh123 were added to A549 and A549/ADR cells for 4 h. The final Rh123 concentration was 1.0 mM. The cells were washed twice with PBS and trypsinized for fluorescence-activated cell sorting (FACS) analyses (Becton–Dickinson, San Jose, CA, USA). In addition, the intracellular content of Rh123 was quantitatively determined according to previously reported methods [28].

To investigate the internalization mechanism, the cells were preincubated with AG-free Hank’s Balanced Salt Solution containing different inhibitors, including two concentrations of AG (2 mM and 10 mM) as GLUT1 transporter inhibitors and 1 μg/mL colchicine, 0.4 μg/mL phenylarsine oxide (PhAsO) and 0.5 μg/mL filipin complex as endocytic inhibitors, at 37 °C for 4 h, followed by incubation with AG-P-SS-P/Rh123 (10 μg/mL) nanomicelles at 37 °C. After incubation, the A549/ADR cells were analyzed by flow cytometry.

#### In vitro cellular targeting assay

In vitro cellular targeting assay were performed to examine the targeting levels in human lung cancer cells as previously described [29]. A549/ADR cells were cultivated in AG-free RPMI-1640 medium supplemented with 10% heat-inactivated FBS and maintained in an incubator containing 5% CO_2_ at 37 °C prior to the experiments. The cells were then incubated with AG-P-SS-P/Rh123 (targeted) or P-SS-P/Rh123 (non-targeted) at 37 °C for 15 min. The final Rh123 concentration was 1.0 mM. To examine AG blocking, free AG (10 mM) was added to the incubation medium prior to the addition of AG-P-SS-P/Rh123 or P-SS-P/Rh123 in a separate set of nanomicelles. The cells were then rinsed with cold PBS three times and fixed with methanol for 20 min; the nuclei of the cells were then stained with Hoechst33342 via the same method used in the cellular uptake assay. Finally, the cells were visualized by confocal laser scanning microscopy (CLSM; Olympus Fluoview-1000, Tokyo, Japan) using imaging software. Cellular uptake was visualized by overlaying the obtained images. Quantitative analysis was performed using ImageJ.

### Cytotoxicity

A549 cells or A549/ADR cells were incubated with fresh culture media containing varying concentrations of P-P, P-SS-P, AG-P-SS-P, PBS, Taxol, P-P/PTX, P-SS-P/PTX, or AG-P-SS-P/PTX at 37 °C for 1 h. The final concentration of PTX was approximately 0–10 μM, and the concentration of the blank nanomicelles was the same as that of the PTX-loaded nanomicelles. Blank culture medium was used as the blank control. After 48 h of incubation, cell viability was measured by proliferation assays [28], with each assay performed in triplicate. Finally, the dose effect curves were created, and the drug concentration that inhibited 50% of cell growth (IC_50_) was calculated by curve fitting the cell viability data to that of the control samples.

### Apoptosis-inducing effect

A549 and A549/ADR cells were seeded in six-well plates (2.5 × 10^5^ cells per well). After incubation for 24 h, the medium was replaced with RPMI 1640 supplemented with the above formulations with different PTX concentrations. The final concentration of PTX was 10 μM. After 24 h of incubation, cell apoptosis was detected using a FITC Annexin V-staining kit and a FACScan flow cytometer according to the standard protocol.

### Western blot analysis

A549 and A549/ADR cells were cultured under 5% CO_2_ at 37 °C for 24 h; Taxol and PTX-loaded nanomicelles were then added to a final concentration of 10 μM, followed by a further 12 h of incubation. Western blot analyses were performed to examine the protein levels in the human lung cancer cells as described previously [3]. Caspase 3, caspase 9, caspase 8, Bcl-2, Bcl-xl, Bax, Bid, and β-actin antibodies were purchased from Cell Signaling Technology (Beverly, MA, USA). The blots were subsequently stripped and reprobed using the β-actin antibody as a loading control.

### In vivo studies

Thirty nude mice weighing 20–22 g (approximately 6–8 weeks of age) were purchased from the animal center at Nanfang Medical University (Guangzhou, China). To induce a tumor, 200 mL of PBS containing 1 × 10^7^ A549/ADR cells was subcutaneously injected into the right flank of the mice. Tumor growth and body weight were recorded every other day. When the volume of the tumor reached approximately 220–230 mm^3^, the animals were randomly divided into six groups: saline, Taxol (10 mg/kg) and AG-P-SS-P, P-P/PTX (10 mg/kg), P-SS-P/PTX (10 mg/kg), and AG-P-SS-P/PTX (10 mg/kg) nanomicelles (n = 5). All formulations were administered to the mice at days 17, 19, 21, 23, 25, and 27 via the tail vein. The mice were then monitored with respect to tumor progression and weight loss every other day, and the tumor volumes were calculated as the length × width^2^/2 (mm^3^). The tumor volume inhibitory rate at day 27 was calculated using the formula Rv = 100% − (V_drug_/V_saline_) × 100%, where V_drug_ is the tumor volume after drug treatment, and V_saline_ is the tumor volume after treatment with physiological saline. In addition, the changes in the body weight of each mouse were monitored throughout treatment to evaluate the possible toxic effects of the therapy. All animal studies were performed using an approved protocol in accordance with the guidelines of the Institutional Animal Care Committee and the Local Veterinary Office and Ethics Committee at Guangzhou Medical University. The animal studies were approved by the Institutional Animal Care Committee and Ethics Committee at Guangzhou Medical University (GZMUC 10-05010). One day after the last injection, the tumor tissues were excised, and the kidneys, spleens, and livers were fixed in 4% formaldehyde for hematoxylin and eosin (HE) assays.

### Statistical analysis

Statistical analyses were performed using GraphPad Prism software. Comparisons were statistically assessed by one-way ANOVA. The data are represented as the means ± standard deviations (SDs).

## Results and discussion

### Synthesis and characterization of AG-P-SS-P

**Fig. 1:**
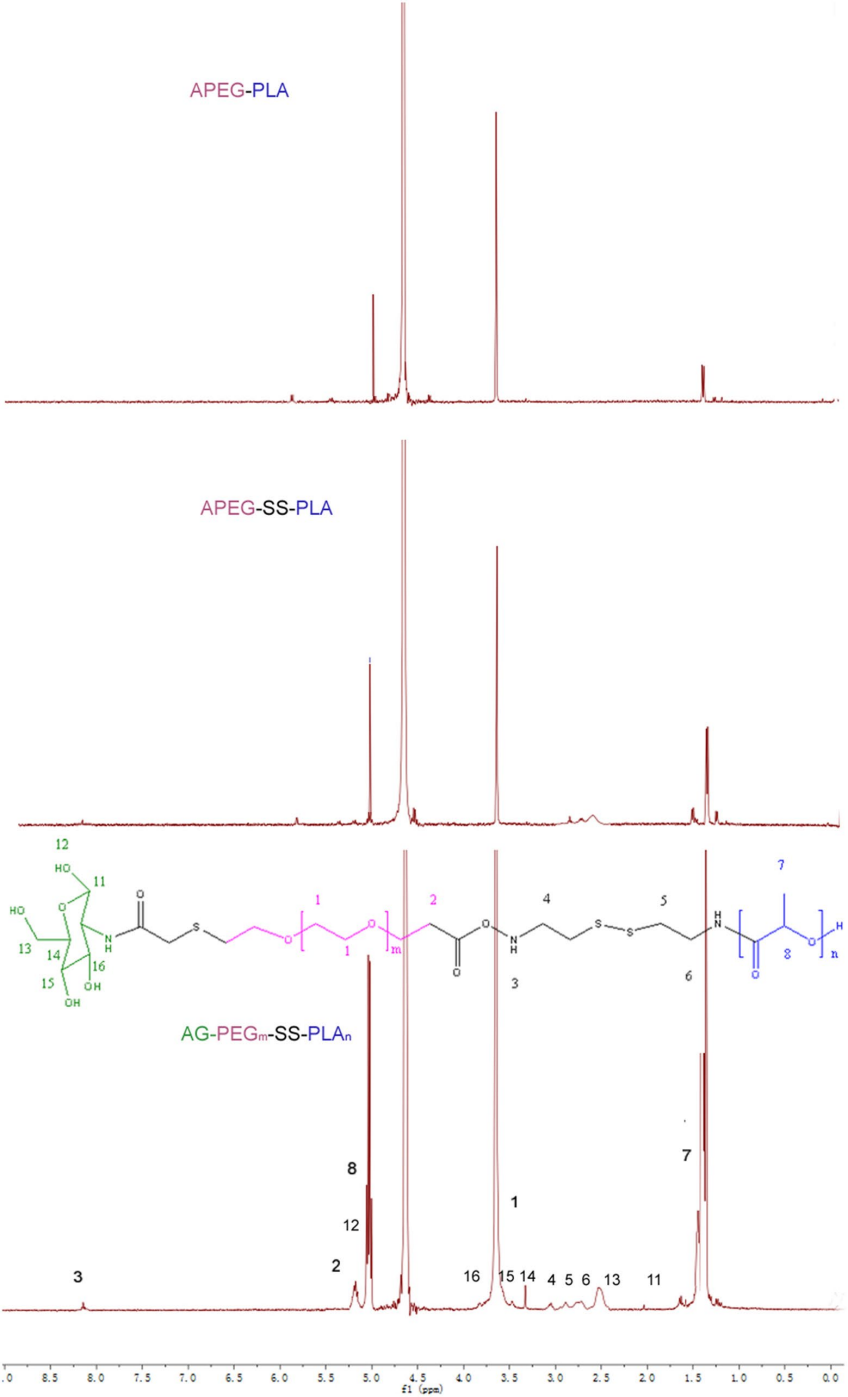
^1^H NMR of P-P, P-SS-P, and AG-P-SS-P

The structure of the diblock copolymer P-SS-P of AG-P-SS-P consists of PEG and PLA blocks bridged by a disulfide bond, and the selective cleavable property of this disulfide bond in response to a redox environment allows structural control of the diblock copolymer. After the intracellular tumor-relevant GSH breaks the disulfide bond, release of the drug inside tumor cells contributes to the disassembly of the hollow structure from the polymer, which helps overcome the MDR of tumor cells by increasing the intracellular accumulation of the drugs. The route used for the synthesis of AG-P-SS-P is shown in Scheme 2. Figure 1 shows the ^1^H-NMR spectrum and complete peak assignments of the AG-P-SS-P copolymer. In the first step, APEG-SS-NH_2_ was synthesized by activating APEG with p-NPC and cystamine·2HCl. To prevent the formation of APEG-SS-APEG as a byproduct, the molar ratio of APEG–NPC to cystamine·2HCl was nearly 1:7. APEG-SS-PLA was then obtained by an amide reaction. In the second step, the distal end of COOH-PEG-SS-PLA, which was transferred from APEG-SS-PLA by the thiol-ene reaction, was conjugated to AG through an amide reaction. A series of characteristic AG peaks were observed, suggesting that AG was successfully conjugated to COOH-PEG-SS-PLA. The molecular weights of the prepared copolymers obtained from gel permeation chromatography (GPC), and their value and the polydispersity index (PDI) were presented in Table 1.

**Table 1:** Molecular weight of the three vectors by GPC

Sample	Mw	Mn	PDI
AG-P-SS-P	19,560	13,216	1.48
P-SS-P	18,855	12,739	1.35
P-P	18,628	14,440	1.29

Polydispersity index (PDI; Mw/Mn) determined by GPC

*GPC* gel permeation chromatography

**Fig. 2:**
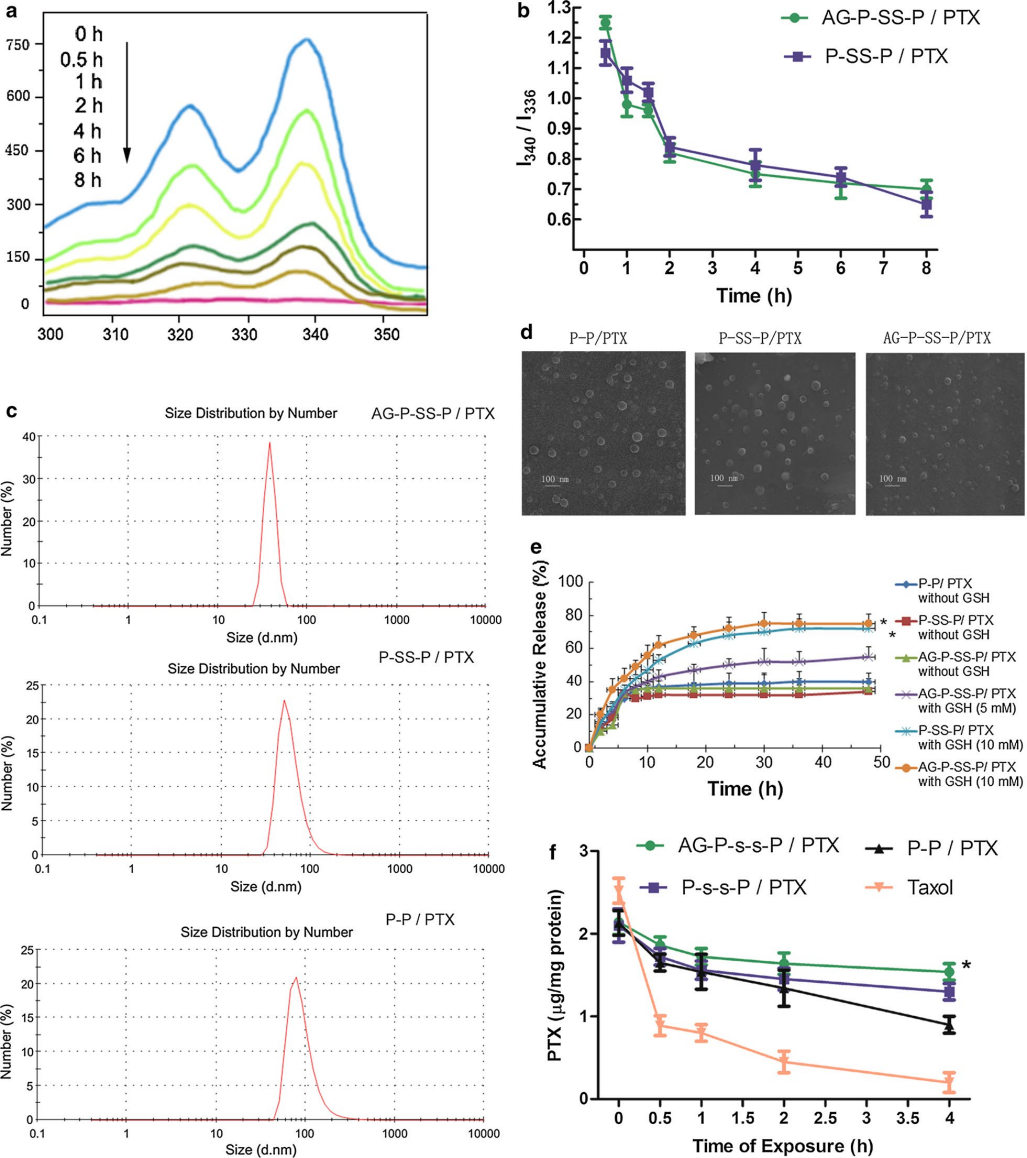
**a** Excitation spectra of pyrene in an AG-P-SS-P solution after incubation with GSH for different time periods (λ_em_ = 373 nm). **b** I340-to-I336 ratio of AG-P-SS-P and P-SS-P after incubation with GSH for different time periods (λ_em_ = 373 nm). DLS measurement (**c**) and TEM images (**d**) of AG-P-SS-P/PTX, P-SS-P/PTX, and P-P/PTX. Scale bar = 100 nm. **e** In vitro PTX release profiles of AG-P-SS-P/PTX, P-SS-P/PTX, and P-P/PTX in PBS (pH 7.4) without GSH at 37 °C; release profile of AG-P-SS-P/PTX in the presence of 5 mM GSH; and release profiles of AG-P-SS-P/PTX and P-SS-P/PTX in the presence of 10 mM GSH. **f** Retention of PTX in A549/ADR cells after preincubation with AG-P-SS-P/PTX, P-SS-P/PTX, P-P/PTX, and Taxol for different time periods. All data are presented as the means ± standard deviations (n = 3); *P < 0.05, compared with P-P/PTX without GSH, P-SS-P/PTX without GSH, AG-P-SS-P/PTX without GSH and AG-P-SS-P/PTX with GSH (5 mM) nanomicelles. *P < 0.05, compared with Taxol, P-P/PTX and P-SS-P/PTX nanomicelles, respectively

To demonstrate the redox-responsive cleavage of the disulfide bond in the polymer, fluorescence measurements using pyrene as a probe were performed at different time intervals after the addition of GSH (10 mM) to a solution of AG-P-SS-P (1.0 mg/mL). The excitation spectral absorption of pyrene shifted from 340.0 to 336.0 nm with increased incubation time (Fig. 2a). However, the same result was not obtained without GSH (data not shown). In addition, the intensity ratio of the bands at 340.0 and 336.0 nm (I340/I336) was plotted against the incubation time, and as shown in Fig. 2b, the I340/I336 ratio significantly decreased with increased incubation time with GSH but did not increase without GSH. This result indicated that the hydrophobic core changed to a polar aqueous solution after the redox-responsive cleavage of the disulfide bond in the polymer, which resulted in the release of pyrene-loaded AG-P-SS-P.

AG-P-SS-P is a disulfide-bridged diblock copolymer of PEG and PLA. The copolymer self-assembled into micellar nanomicelles in aqueous solution and then encapsulated PTX in its core, generating nanomicelles with an average diameter of 85 ± 2.52 nm. Nanomicelles of P-P (90 ± 2.40 nm) and P-SS-P (87 ± 2.50 nm) with similar particle sizes and size distributions were developed for comparison purposes (Fig. 2c), and these encapsulated PTX in their hydrophobic cores at comparable drug-loading contents (∼ 8% w/w). Furthermore, TEM images showed that all of the nanomicelles were spheroidal in shape and homogeneous in size (Fig. 2d). The AG-modified nanomicelles were much closer together than the unmodified P-SS-P nanomicelles, and this disparity might be due to the enhanced hydrophilicity of the nanomicelle surface after the incorporation of AG. In addition, after incubation with and without medium containing 10% FBS, the sizes of the three PTX-loaded nanomicelles were maintained for 96 h, indicating the excellent stability of the three nanomicelles (Additional file 1: Figure S2A, B). Information on the nanomicelles is summarized in Table 2.

**Table 2:** Characterization of nanomicelles

	Blank nanomicelles	P-P/PTX nanomicelles	P-SS-P/PTX nanomicelles	AG-P-SS-P/PTX nanomicelles
Particle size (nm)	74 ± 1.80	90 ± 2.40	87 ± 2.50	75 ± 2.52
Zeta potential (mV)	− 1.65 ± 0.54	− 2.56 ± 1.35	− 2.68 ± 0.75	− 0.25 ± 1.55
PDI	0.196 ± 0.003	0.221 ± 0.004	0.244 ± 0.005	0.245 ± 0.005
Encapsulation (%)	–	78.45 ± 4.34	80.21 ± 3.43	84.21 ± 3.75
DLC (%)	–	7.8 ± 0.6	7.9 ± 0.6	8.2 ± 0.7

### Intracellular release and retention of redox-responsive nanomicelles in MDR cancer cells

To demonstrate the intracellular PTX release of every formulation, HPLC analyses were performed. As shown in Fig. 2e, an obvious initial burst release of PTX was observed for all of the formulations. In the absence of GSH, more than 30% of the PTX was released from drug-loaded AG-P-SS-P at 5 h, and 40% was released at 48 h. However, in the presence of 5 mM GSH, the cumulative release of PTX from AG-P-SS-P/PTX was increased to 53% at 48 h, and in the presence of 10 mM GSH, the cumulative release increased to 78% at 48 h, indicating that the release profiles are dependent on the GSH concentration. In addition, a marked difference was not observed between P-SS-P and AG-P-SS-P with or without GSH. The addition of GSH to the release medium of AG-P-SS-P/PTX significantly accelerated the release rate (Additional file 1: Figure S3), an effect that is attributed to the cleavage of the disulfide bonds [30]. The results suggested that drug release from AG-P-SS-P/PTX nanomicelles was accelerated in the medium with the reducing agent and triggered under conditions mimicking the intracellular environment.

Overexpression of P-gp protein is responsible for the release of intracellular PTX; [31] thus, the retention of AG-P-SS-P/PTX nanomicelles in A549/ADR cells was assayed. To avoid an initial concentration effect, 5.5 μg/mL PTX-loaded nanomicelles or 42 μg/mL free PTX, comparable to cellular PTX levels, were incubated with A549/ADR cells for 4 h. The cells were then washed with PBS and incubated in fresh medium for different periods of time before the intracellular PTX concentrations were quantitatively examined by HPLC. After 4 h of incubation, 71.9 ± 2.1% of the PTX was retained in cells incubated with the AG-P-SS-P/PTX nanomicelles, a significantly higher amount than the internalized PTX that was retained in the A549/ADR cells incubated with P-SS-P/PTX (57.1 ± 1.7%), P-P/PTX (40.2 ± 2.3%), and Taxol (9.4 ± 0.14%), suggesting that the AG-P-SS-P/PTX nanomicelles have a significantly lower rate of drug efflux (Fig. 2f). These results indicated that the combination of GLUT-1 media and redox-responsive nanomicelles could bypass the P-gp-mediated efflux of PTX and yield enhanced intracellular accumulation and retention of PTX in MDR tumor cells.

### Uptake of AG-P-SS-P by lung cancer cells

**Fig. 3:**
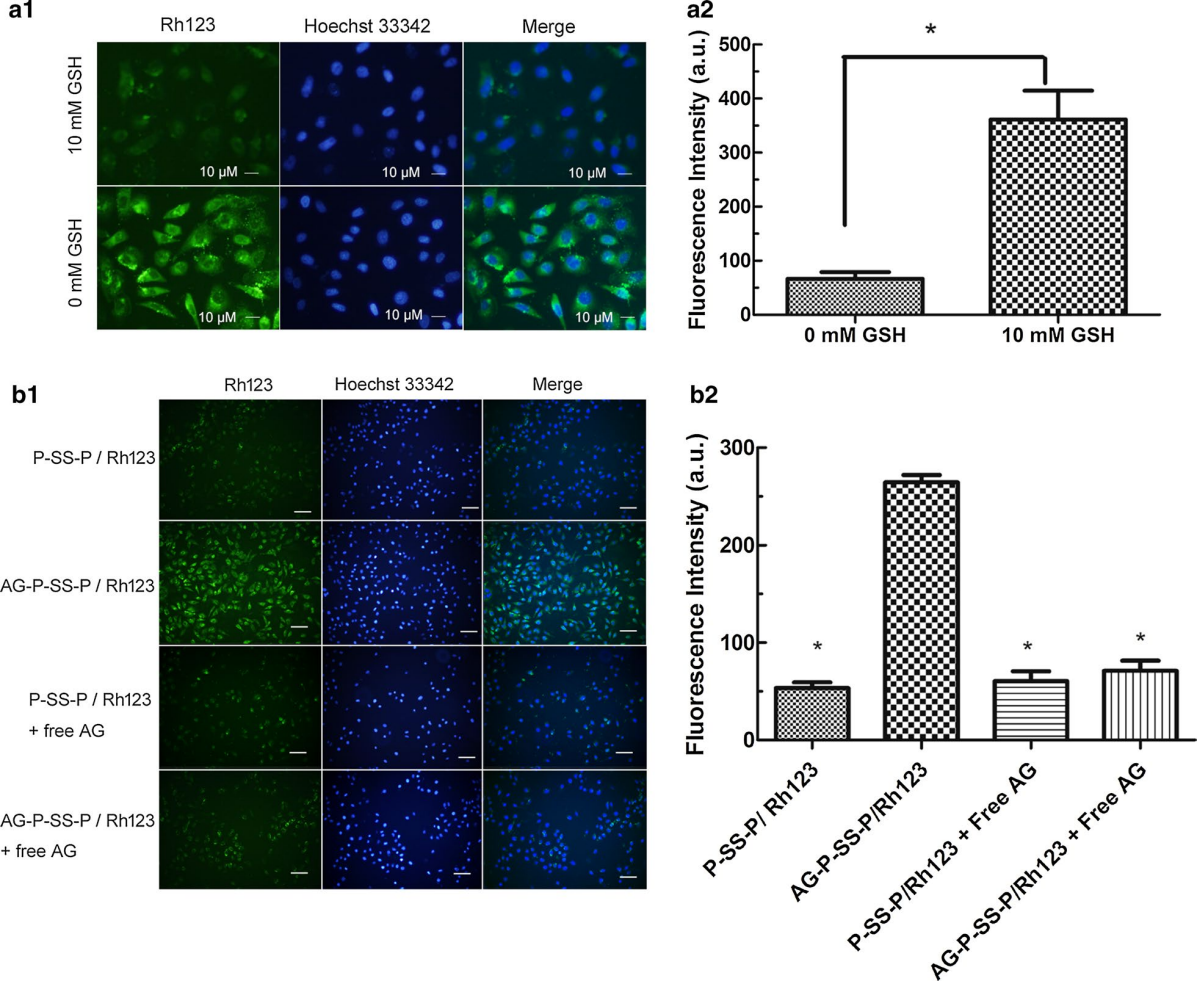
**a** The cellular uptake of AG-P-SS-P/Rh123 with 0 and 10 mM GSH by A549/ADR cells using laser scanning confocal microscopy (×400). **b1** CLSM images of A549/ADR cells after 30 min of incubation with P-SS-P/Rh123 (non-targeted) and AG-P-SS-P/Rh123 (targeted) (×200). Free AG (10 mM) was used to block GLUT1 binding before adding AG-P-SS-P/Rh123. **b2** Fluorescence intensity of Rh123-loaded P-SS-P (non-targeted) and AG-P-SS-P (targeted) in A549/ADR cells. Quantitative analysis was performed with ImageJ. *P < 0.05, compared with 10 mM GSH. *P < 0.05, compared with AG-P-SS-P/Rh123 nanomicelles, respectively. (1) Image of CLSM; (2) Fluorescence intensity of image

**Fig. 4:**
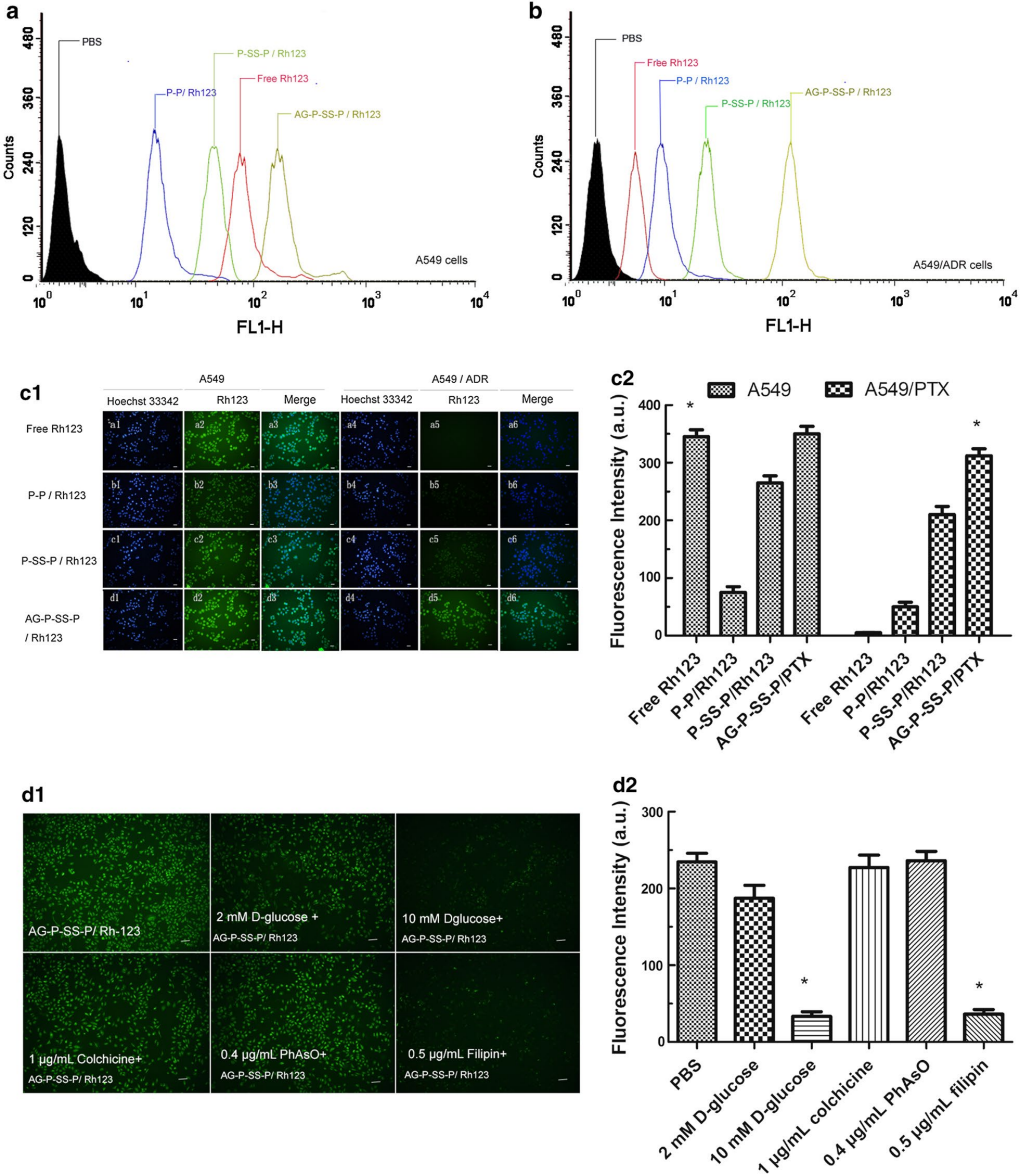
Flow cytometry analyses of A549 (**a**) and A549/ADR (**b**) cells following a 2-h incubation with free Rh123, P-P/Rh123, P-SS-P/Rh123, or AG-P-SS-P/Rh123. **c** Laser scanning confocal microscopy-based comparison of the cellular uptake of Rh123 from different formulations by A549 and A549/ADR cells (scale bar, 30 mm). **d** Analysis of the pathway responsible for the uptake of AG-P-SS-P/Rh123 nanomicelles by A549 cells. This analysis was performed 30 min after incubation. The cells were blocked with different inhibitors: 2 mM AG, 10 mM AG, 1 μg/mL colchicine, 0.4 μg/mL PhAsO, or 0.5 μg/mL filipin. Green, Rh123 (scale bar, 50 mm). *P < 0.05, compared with Free Rh123 in A549/PTX cells, respectively. *P < 0.05, compared with PBS, respectively. (1) Image of CLSM; (2) fluorescence intensity of image

To verify whether the obtained redox-sensitive nanomicelles could overcome drug resistance, the redox-responsive function of the AG-P-SS-P nanomicelles was evaluated in A549/ADR cells. As shown in Fig. 3a, the intensity of AG-P-SS-P/Rh123 fluorescence with 10 mM GSH was significantly stronger than that in 0 mM GSH. To determine whether GLUT-1-targeted nanomicelles can overcome drug resistance, the targeted function of the AG-P-SS-P nanomicelles was also evaluated in A549/ADR cells. Figure 3b shows that the fluorescence intensity of targeted AG-P-SS-P/Rh123 nanomicelles in the cytoplasm was increased 4.9-fold higher than the non-targeted P-SS-P nanomicelles in the AG-free group. However, when free AG (10 mM) was added to the A549/ADR cells before AG-P-SS-P/Rh123, the cellular uptake efficiency of the targeted AG-P-SS-P/Rh123 decreased substantially due to the competitive binding of free AG and GLUT-1, suggesting that AG-P-SS-P nanomicelles have a significant active targeting effect. Subsequently, flow cytometry analysis of the intracellular Rh123 fluorescence-loaded nanomicelles was performed after incubation for 2 h. As shown in Fig. 4a, b, the intensity of the intracellular fluorescence of AG-P-SS-P/Rh123 was significantly stronger than that of P-SS-P/Rh123, P-P/Rh123, and free Rh123 in both A549 and A549/ADR cells. Confocal microscopy images of A549 or A549/ADR cells obtained 2 h after the addition of free Rh123, P-P/Rh123, P-SS-P/Rh123, or AG-P-SS-P/Rh123 nanomicelles showed similar results (Fig. 4c). The highly hydrophilic free Rh123 readily diffused into the A549 cells and caused obvious cellular accumulation. However, the fluorescence intensity of free Rh123 was minimal in the resistant A549/ADR cells. In contrast, overlaid images of the AG-P-SS-P/Rh123 nanomicelles in the cell cytoplasm displayed more intense Rh123 green fluorescence than did the P-SS-P/Rh123 nanomicelles, P-P/Rh123 nanomicelles or free Rh123 in both A549 and A549/ADR cells. Importantly, the intense green fluorescence of AG-P-SS-P/Rh123 in A549/ADR cells was similar to that in A549 cells. These results further indicated that the tumor-targeting and redox-sensitive AG-P-SS-P nanomicelles can override drug resistance.

**Fig. 5:**
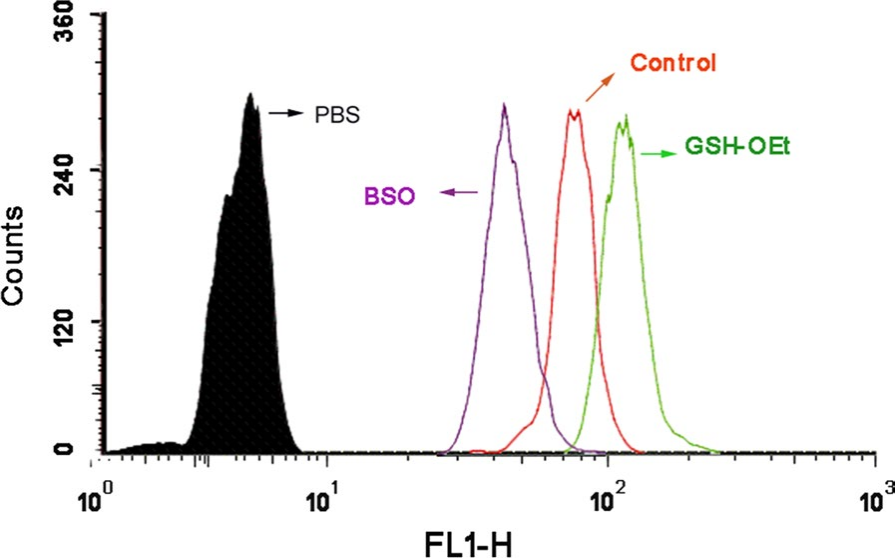
Flow cytometry analyses of A549/ADR cells after incubation with AG-P-SS-P/Rh123. The A549/ADR cells were precultured with BSO or GSH-OEt. *P < 0.05, compared with Free Rh123 in A549/PTX cells, respectively. *P < 0.05, compared with PBS, respectively. 1, image of CLSM; 2, Fluorescence intensity of image

### Mechanism of endocytosis

To investigate the possible internalization mechanism of the AG-P-SS-P/Rh123 nanomicelles, we inhibited cellular uptake using various inhibitors, including AG (which blocks GLUT1), colchicine (which blocks macropinocytosis), phenylarsine oxide (which blocks the clathrin-dependent pathway) and filipin complex (which blocks the caveolae-mediated pathway) (Fig. 4d). Notably, a minor effect on the internalization of AG-P-SS-P/Rh123 nanomicelles was observed with a low concentration of AG (2 mM). However, this inhibition effect was enhanced by increasing the concentration of AG to a high level (10 mM). Among the three endocytic inhibitors, the filipin complex exhibited the most significant inhibition effect, indicating that caveolae-mediated endocytosis, which is different than the conventional receptor-mediated clathrin-dependent endosome route, was the main endocytosis pathway of AG-P-SS-P/Rh123 [32, 33]. Based on these results, we hypothesized that AG is the agent mainly responsible for actively recognizing and binding to lung cancer cells via GLUT1 and that caveolin-dependent endocytosis is the mechanism mainly responsible for the internalization of the nanomicelles.

To investigate whether the redox-responsive nanomicelles can achieve rapid intracellular PTX release in the presence of a high intracellular GSH concentration, A549/ADR cells were incubated with glutathione monoester (GSH-OEt) for 2 h or (buthionine sulfoximine) BSO for 12 h to downregulate and upregulate their intracellular GSH concentrations, respectively [34]. Flow cytometry analysis was then performed to assess intracellular Rh123 fluorescence after incubation for 4 h. Figure 5 shows that the fluorescence intensity of intracellular PTX after pretreatment with BSO was significantly weaker relative to that of the control, whereas the strongest intracellular Rh123 intensity was observed after pretreatment with GSH-OEt. The release of Rh123 from the redox-responsive micelles, which were quenched after encapsulation, was enhanced by the increased intracellular GSH concentration obtained after pretreatment with GSH-OEt, which led to enhanced intracellular Rh123 fluorescence. However, the decreased intracellular GSH concentration obtained after pretreatment with BSO resulted in a slower Rh123 release rate and a subsequently weaker intracellular fluorescence.

### Inhibitory effect on resistant lung cancer cells

**Fig. 6:**
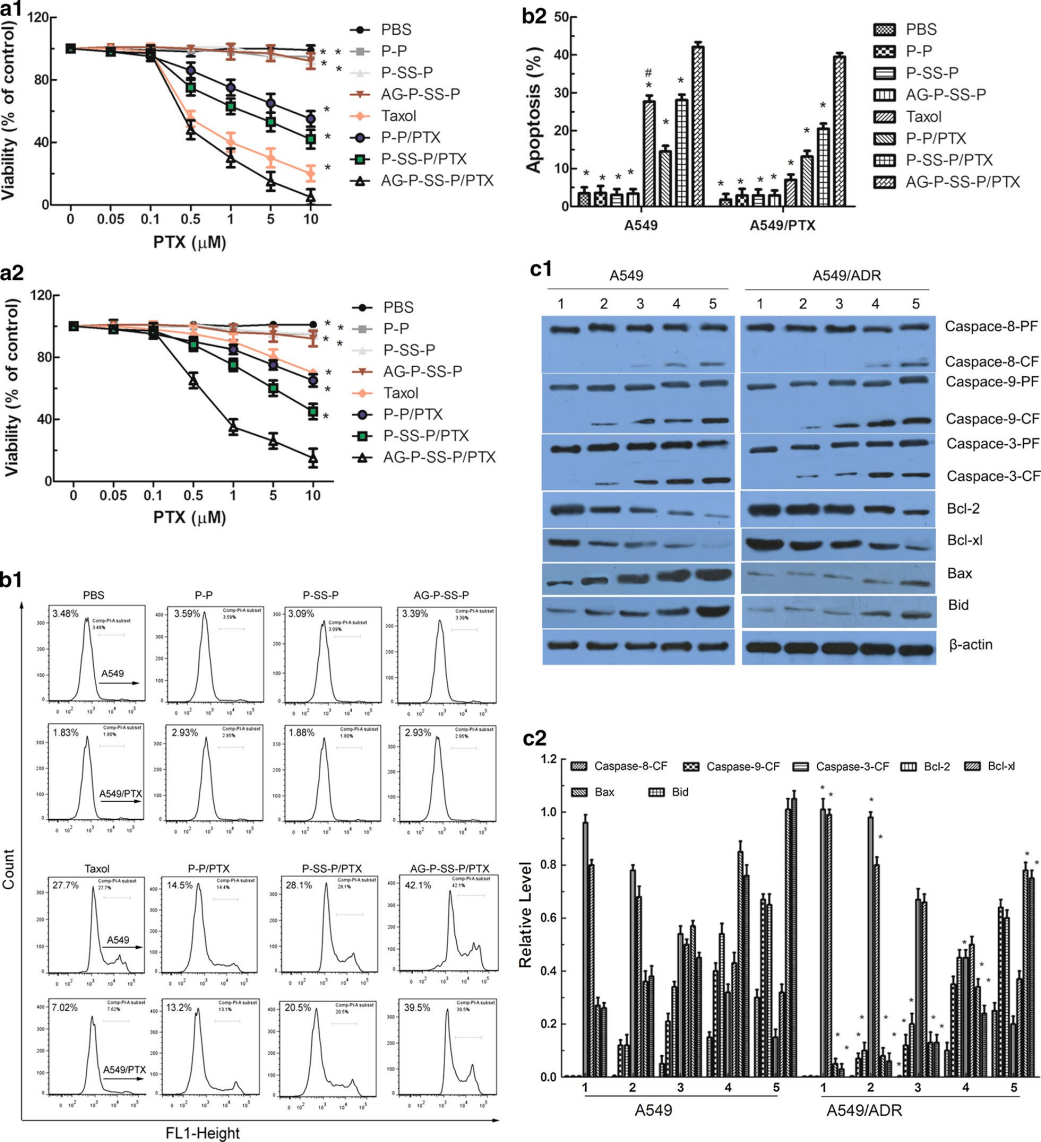
**a** Viability of A549 (**a1**) and A549/ADR (**a2**) cells cultured with PTX-loaded nanomicelles in comparison with that of Taxol at the same PTX dose for 48 h. **b** Cell apoptosis rate detected by flow cytometry. A549 and A549/ADR cells were treated with different formulations that contained a total PTX concentration of 10 µM for 24 h. **c** Proteins involved in the apoptosis signaling pathways in A549 and A549/ADR cells as determined by Western blotting (**c1**). (1) Control (PBS); (2) Taxol; (3) P-P/PTX; (4) P-SS-P/PTX; and (5) AG-P-SS-P/PTX nanomicelles. Activity ratios of caspase-3 and caspase-9 and expression ratios of the pro-apoptotic proteins Bax and Bid and the anti-apoptotic proteins Bcl-2 and Bcl-xl in A549 and A549/ADR cells after incubation with the various formulations. β-actin was also assessed by Western blotting. All protein levels were quantified densitometrically and normalized to β-actin (**c2**). All data are presented as the means ± standard deviations (n = 3); (1) image of western blot; (2) grey level of western blot. *P < 0.05, compared with AG-P-SS-P/PTX nanomicelles. ^#^P < 0.05, compared with A549 cells

To evaluate the cytotoxicity of AG-P-SS-P/PTX nanomicelles, cell proliferation assays were performed using A549 and A549/ADR cells. Figure 6a shows that cell viability was inhibited more significantly by AG-P-SS-P/PTX nanomicelles than by P-SS-P/PTX, P-P/PTX, and Taxol. Although taxol caused lower viability in A549/ADR cells than in A549 cells, AG-P-SS-P/PTX nanomicelles significantly inhibited the viability of A549/ADR cells. Importantly, the IC_50_ values after 48 h of incubation with AG-P-SS-P/PTX in A549/ADR (3.51 µM) cells was close to that in A549 (2.14 µM) cells, indicating that AG-P-SS-P/PTX nanomicelles can overcome the resistance of A549/ADR (Table 3). In addition, the cytotoxicity of drug-free nanomicelles (for example, AG-P-SS-P, P-SS-P, and P-P), were also assayed in lung cancer cells, and cell cytotoxicity was hardly observed, indicating that these vectors are biocompatible and non-toxic to tissues and cells.

**Table 3:** IC50 values of Taxol and every PTX loaded nanomicelles on A549 and A549/PTX cells after 48 h incubation (n = 5)

IC50 (µM)				
Cells	Taxol	P-P/PTX	P-SS-P/PTX	AG-P-SS-P/PTX
A549	3.28	9.70	7.36	2.14
A549/PTX	12.49	11.9	8.25	3.51

### In vitro apoptosis-inducing effect

To examine whether the encapsulation of PTX in glucosylated and redox-responsive nanomicelles induces cell apoptosis, an Annexin V-FITC Apoptosis Detection kit was used to stain the cells, and the percentage of early period cell apoptosis was determined by flow cytometry. Figure 6b depicts the apoptosis-inducing effects of PBS, Taxol, P-P/PTX nanomicelles, P-SS-P/PTX nanomicelles, and AG-P-SS-P/PTX nanomicelles; the percentages of apoptosis induction detected in A549/ADR cells (3.77, 9.75, 21.15, 23.56, and 35.36%, respectively) were similar to those observed in A549 cells (4.72, 22.54, 23.36, 30.30, and 37.48%, respectively), except for the apoptosis rate of Taxol, which was significantly lower in A549/ADR cells than in A549 cells. AG-P-SS-P/PTX nanomicelles reduced the apoptosis induction differences between A549 and A549/ADR cells. These findings further indicated that the AG-P-SS-P/PTX nanomicelles could effectively overcome drug resistance. In addition, the apoptosis-inducing effect of drug-free nanomicelles (for example, AG-P-SS-P, P-SS-P, and P-P), were also assayed in lung cancer cells, and apoptosis-inducing effect cytotoxicity was hardly observed, indicating that these vectors are biocompatible and non-toxic to tissues and cells.

### Apoptosis signaling pathways

#### Caspase activities

To investigate the mechanism through which the AG-P-SS-P/PTX nanomicelles induce lung cancer cell apoptosis, we analyzed whether treatment with the AG-P-SS-P/PTX nanomicelles activated caspases, which are key executioners of apoptosis [35]. As shown in Fig. 6c, the application of the AG-P-SS-P/PTX nanomicelles significantly enhanced the activities of caspase-3 and caspase-9 in both A549 and A549/ADR cells and slightly changed the activity of caspase-8, which indicated that the intrinsic apoptosis pathway is primarily involved in the observed apoptosis. Interestingly, the expression of caspase-3 and caspase-9 in response to the functional PTX nanomicelles was nearly equivalent in A549 and A549/ADR cells, suggesting that drug resistance was overcome by the functional PTX nanomicelles.

#### Expression of Bcl-2 family proteins

Cancer apoptosis is controlled by the Bcl-2 family [36]. Therapeutic strategies that target Bcl-2 represent a promising approach for treating many types of cancers [37] because increased expression of the pro-apoptotic Bcl-2 family of proteins promotes cancer cell apoptosis, whereas increased expression of proteins belonging to the anti-apoptotic Bcl-2 family promotes cancer cell survival [38]. In the current study, the AG-P-SS-P/PTX nanomicelles enhanced pro-apoptotic protein (Bax and Bid) expression and reduced anti-apoptotic protein (Bcl-2 and Bcl-xl) expression in A549 and A549/ADR cells (Fig. 6c). The AG-P-SS-P/PTX nanomicelles showed an outcome effect relative to the PTX, P-P/PTX nanomicelles, and P-SS-P/PTX nanomicelles, indicating that the AG-P-SS-P/PTX nanomicelles could enhance the apoptosis of drug-resistant A549/ADR cells by activating pro-apoptotic proteins and suppressing anti-apoptotic proteins.

### Anticancer efficacy in resistant human lung cancer xenografts

**Fig. 7:**
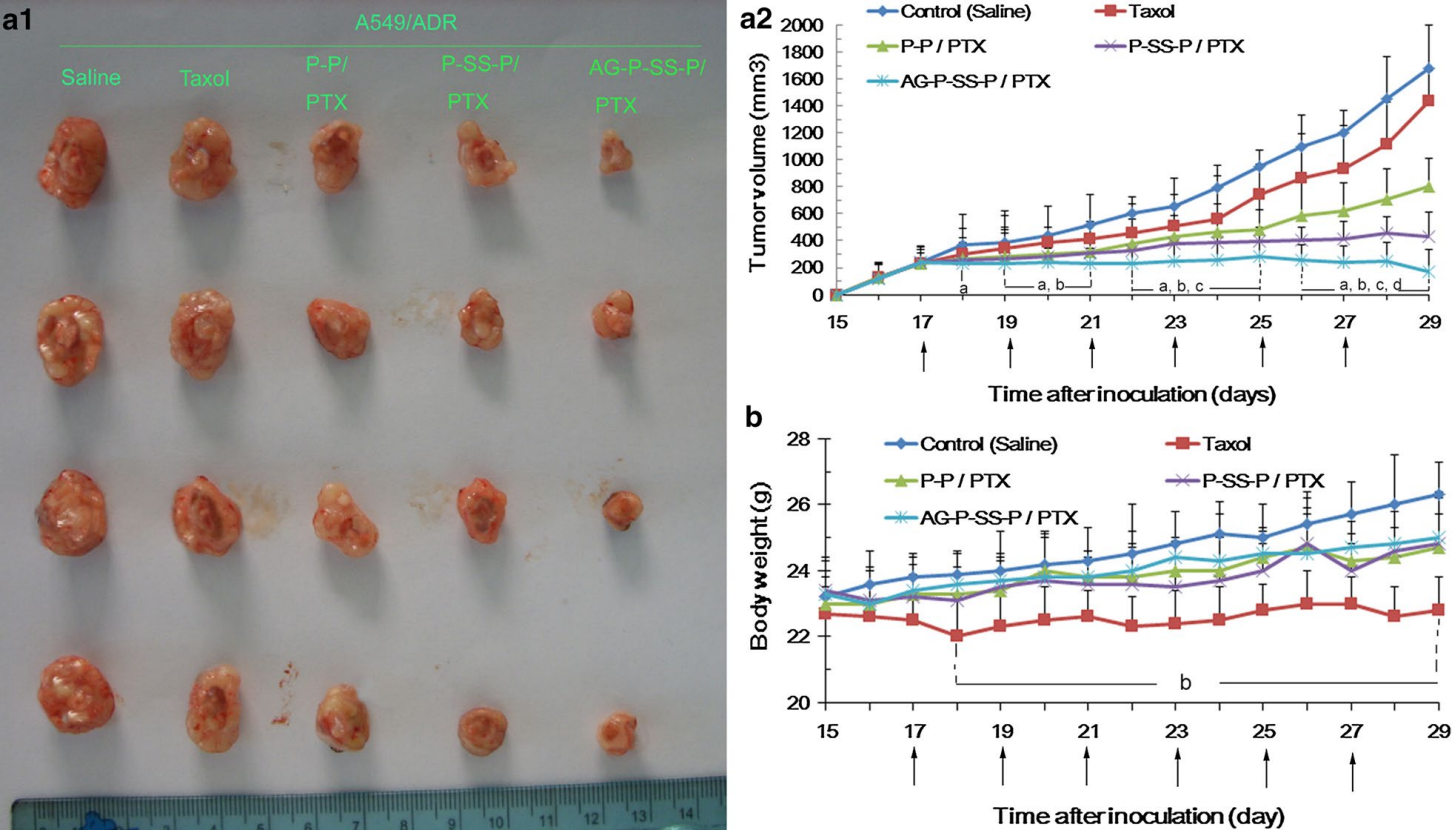
Tumor images (**a1**) and tumor growth inhibition graph (**a2**) for a murine model with A549/ADR xenografts after intravenous injection with the different formulations. **b** Body weight changes of the tumor-bearing mice after treatment with the various formulations. The arrow indicates the day of drug administration. The data are presented as the means ± standard deviations (n = 5); ^a^P < 0.05 compared with the control; ^b^P < 0.05 compared with Taxol; ^c^P < 0.05 compared with P-P/PTX; and ^d^P < 0.05 compared with P-SS-P/PTX nanomicelles

The analysis of different formulations with the same dose of PTX using resistant A549/ADR-xenografted nude mice revealed that the most significant antitumor activity was obtained with the AG-P-SS-P/PTX nanomicelles, and tumor growth was markedly inhibited with this formulation (Fig. 7a). As shown in this study, the uptake permeability of resistant tumors, which enhances the cytotoxic effects on the drug-resistant cancer cells, was enhanced by the addition of a disulfide bond and AG to the functional PTX nanomicelles. In addition, compared with Taxol, AG-P-SS-P nanomicelles do not cause an appreciable reduction in body weight (Fig. 7b). The main reasons for this finding are the suitable particle size of the AG-P-SS-P nanomicelles, which allows greater accumulation of PTX in tumor tissues due to the enhanced permeability and retention (EPR) effect, and the increased uptake of the AG-P-SS-P nanomicelles, which enhances their cytotoxicity in drug-resistant lung cancer cells. Additionally, the lack of toxicity observed in the various treatment groups (Additional file 1: Figure S4A) indicated that the use of PEGylated materials in the AG-P-SS-P/PTX nanomicelles improved the pharmacokinetic profile of PTX [39, 40], which ultimately resulted in higher accumulation in tumors. In addition, we did not observe any differences in the anticancer effects or toxicity to normal tissue between AG-P-SS-P nanomicelles and saline, indicating that AG-P-SS-P have no effect on A549/ADR-induced tumor-bearing mice (Additional file 1: Figure S4A–D).

## Conclusions

This study focused on aminoglucose-functionalized, redox-responsive copolymer AG-P-SS-P vesicles as a novel hydrophilic anticancer drug delivery system for overcoming MDR. The administration of these vesicles resulted in nanomicelle disassembly and rapid drug release. Incubation of the nanomicelles with drug-resistant cells significantly enhanced the intracellular accumulation and retention of PTX because the nanomicelles were internalized by the resistant cells through the endocytosis pathway, thereby avoiding P-gp-mediated drug efflux. AG modification on the surface of the nanomicelles significantly facilitated their specific uptake by cancer cells via GLUT protein-mediated endocytosis. Moreover, intracellular GSH also induced rapid intracellular release. Therefore, the PTX-loaded dual-function nanomicelles more effectively inhibited the proliferation of drug-resistant A549/ADR cells in vitro and in vivo. Overall, this study demonstrates that AG-P-SS-P nanomicelles show great potential for overcoming drug resistance in cancer therapy.

## Additional file

**Additional file 1.** Additional figures.

## Abbreviations

MDR: multiple-drug resistance
GLUT-1: glucose transporter-1
GSH: glutathione
AG: aminoglucose
P-gp: P-glycoprotein
APEG: allyloxy polyethylene glycol
p-NPC: *p*-nitrophenyl chloroformate
Rh123: rhodamine 123
THF: anhydrous tetrahydrofuran
FBS: fetal bovine serum
TEM: transmission electron microscope
DCM: dichloromethane
DLC: drug-loading capacity
DLE: drug-loading efficiency
CDDP: cisplatin
PhAsO: phenylarsine oxide
