# Supplementary material for: Aminoglucose-functionalized, redox-responsive polymer nanomicelles for overcoming chemoresistance in lung cancer cells
Source: J Nanobiotechnology. 2017 Nov 28;15:87. doi: 10.1186/s12951-017-0316-z (PMC5704373; doi:10.1186/s12951-017-0316-z)
Supplement: Supplementary file 1 — Additional file 1. Additional figures. [file 12951_2017_316_MOESM1_ESM.docx]

Additional Materials

**
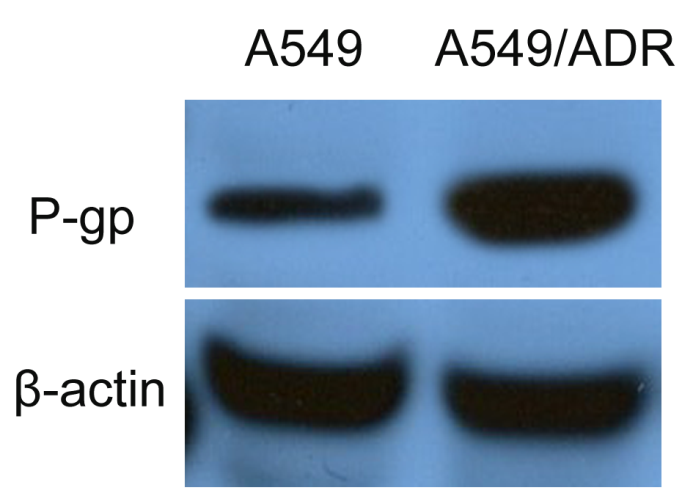
**

A

**
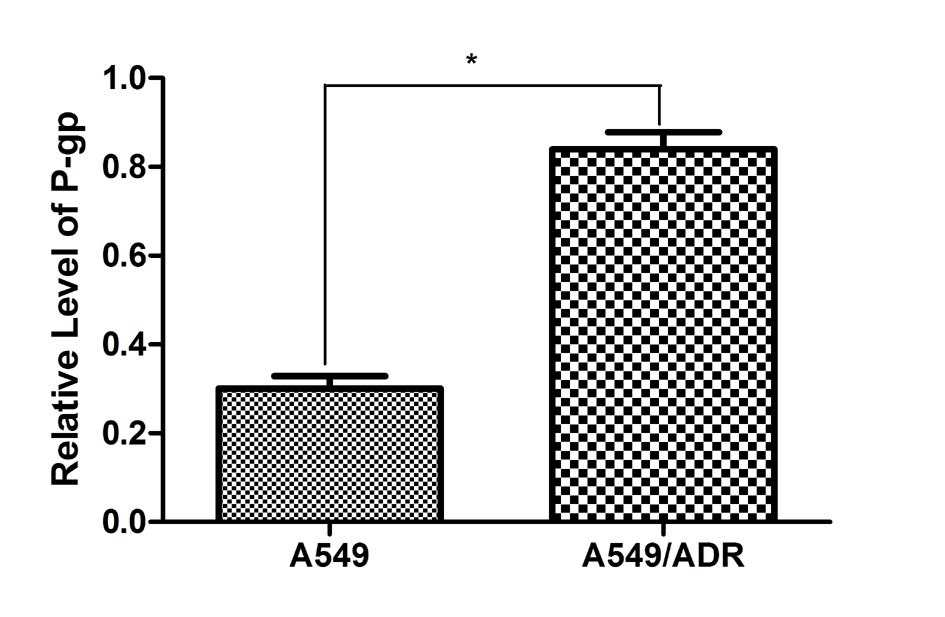
**

B

**Figure S1.** Western blot detection of P-gp in A549 and A549/ADR cells. A, image of western blot; B, grey level of western blot. *p < 0.001, A549 vs A549/ADR. All results are tested for three independent experiments.


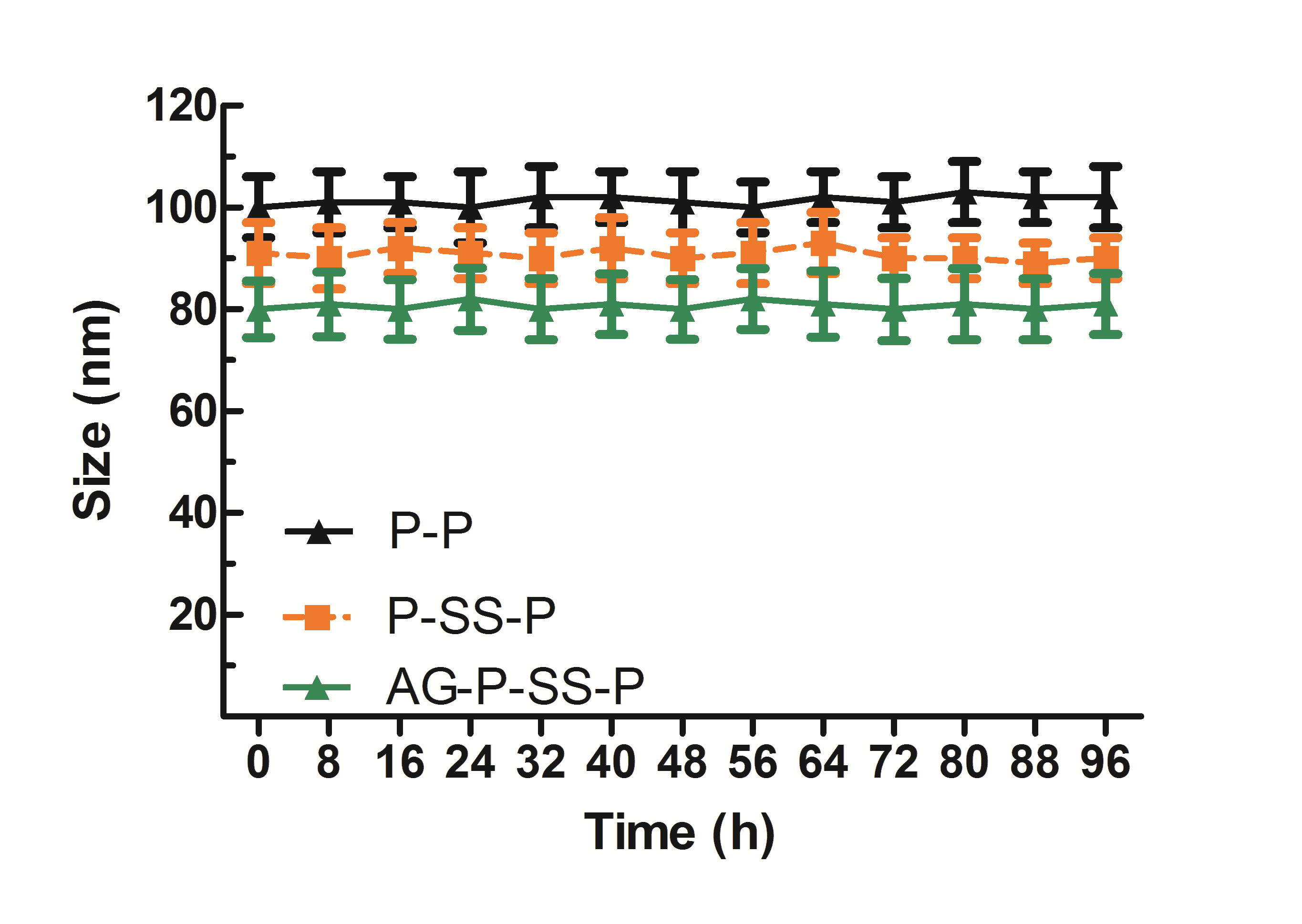

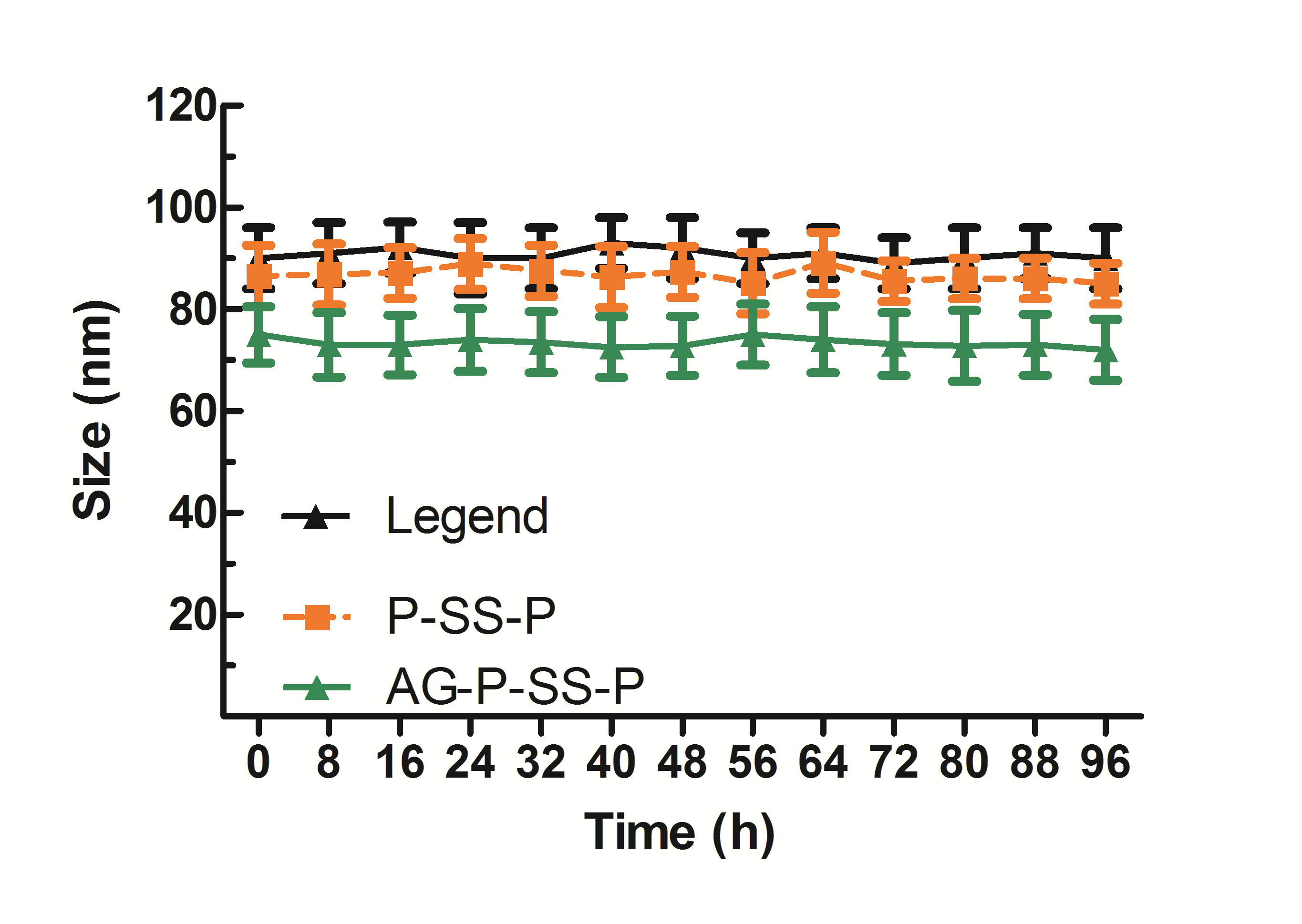


**Figure S2.** Effect of incubation in serum (10%)-containing DMEM media (Left on panel) and PBS (Right on panel) on the particle size of the nanomicelles.

**
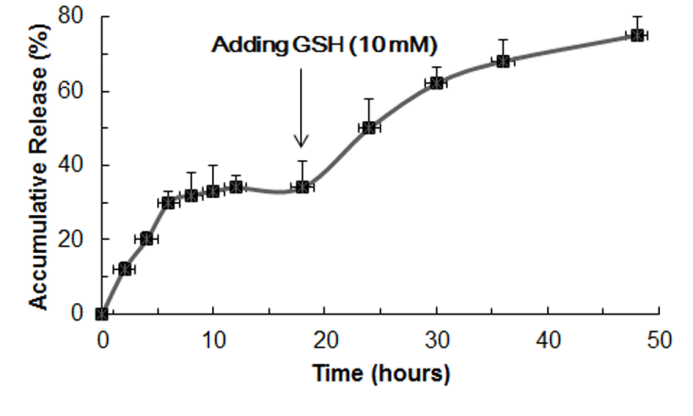
**

Figure S3. In vitro PTX release profile AG-P-SS-P / PTX in PBS (pH 7.4) without GSH at 37 °C for 20 h, followed by adding GSH (10 mM) into the solution and monitoring the release behavior for additional 28 h. Data are presented as the average ± standard deviation (n = 3)


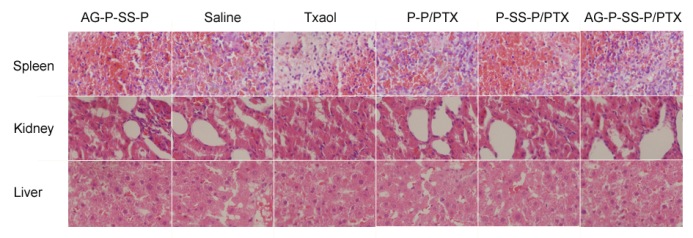


**Fig.S4A**

**
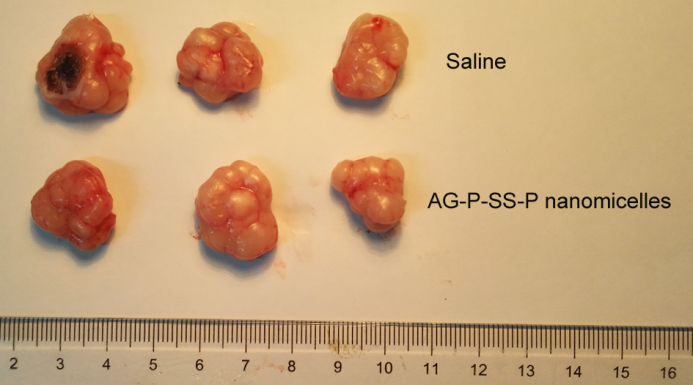
**

**Fig.S4B**

**
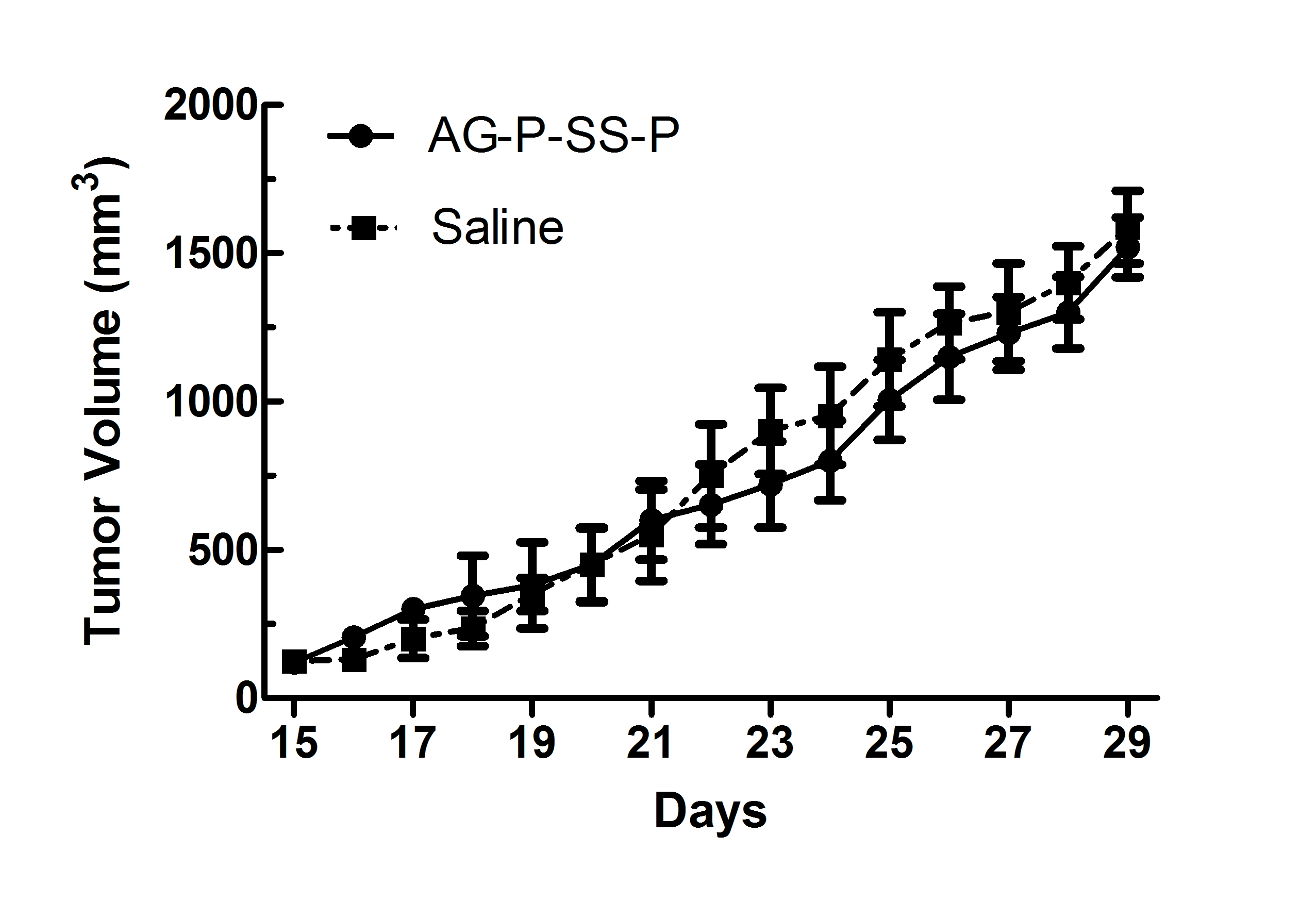
**

**Fig.S4C**

**
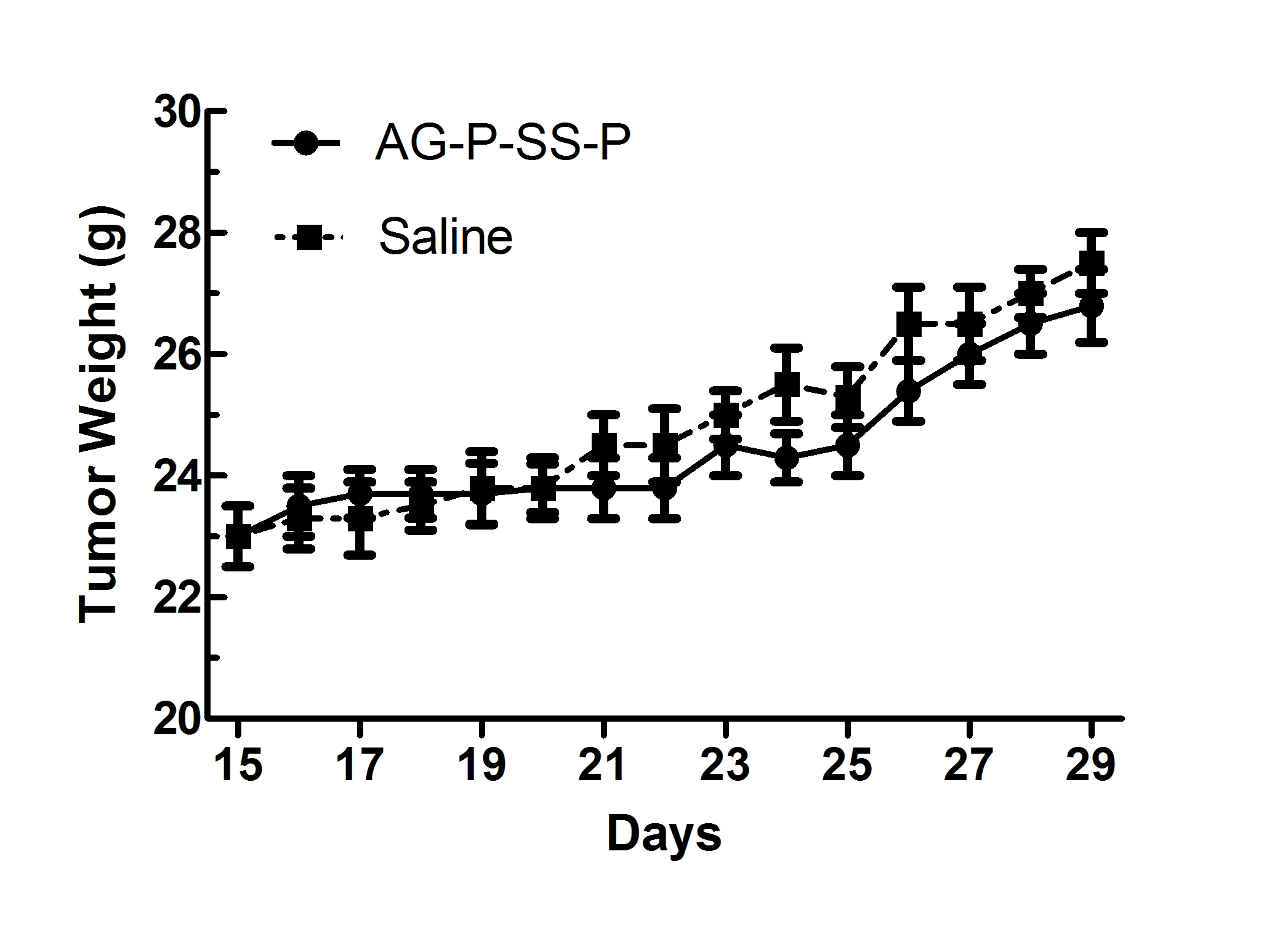
**

**Fig.S4D**

**Figure S4.** (A) Images of HE staining did not detect any toxic response in liver, kidney, and spleen in tumor-bearing mice 29 days after tail vein injection of the various complexes(X 400). Tumor images (B) and tumor growth inhibition graph (C) for a murine model with A549/ADR xenografts after intravenous injection with saline and AG-P-SS-P nanomicelles. (D) Body weight changes of the tumor-bearing mice after treatment with saline and AG-P-SS-P nanomicelles.
